# Effects of Proactive Social Distancing on COVID-19 Outbreaks in 58 Cities, China

**DOI:** 10.3201/eid2609.201932

**Published:** 2020-09

**Authors:** Zhanwei Du, Xiaoke Xu, Lin Wang, Spencer J. Fox, Benjamin J. Cowling, Alison P. Galvani, Lauren Ancel Meyers

**Affiliations:** The University of Texas at Austin, Austin, Texas, USA (Z. Du, S.J. Fox, L.A. Meyers);; Dalian Minzu University, Dalian, China (X. Xu); University of Cambridge, Cambridge, UK (L. Wang);; The University of Hong Kong, Hong Kong, China (B.J. Cowling);; Center for Infectious Disease Modeling and Analysis, Yale School of Public Health, New Haven, Connecticut, USA (A.P. Galvani);; Santa Fe Institute, Santa Fe, New Mexico, USA (L.A. Meyers)

**Keywords:** COVID-19, SARS-CoV-2, severe acute respiratory syndrome coronavirus 2, viruses, respiratory infections, zoonoses, coronavirus disease, epidemiology, reproduction number, nonpharmaceutical interventions

## Abstract

Cities across China implemented stringent social distancing measures in early 2020 to curb coronavirus disease outbreaks. We estimated the speed with which these measures contained transmission in cities. A 1-day delay in implementing social distancing resulted in a containment delay of 2.41 (95% CI 0.97–3.86) days.

On December 31, 2019, a cluster of atypical pneumonia in Wuhan, China, was reported to the regional office of the World Health Organization (WHO). Its etiology was later identified as the novel severe acute respiratory syndrome coronavirus 2 (SARS-CoV-2). Coronavirus disease (COVID-19) spread rapidly across China and internationally (*1*); as of April 9, 2020, a total of 1,436,198 confirmed cases and 85,522 deaths had been reported in 209 countries (*2*). In the absence of pharmaceutical prophylactic options, the primary means of COVID-19 control are social distancing interventions, including school closures, work restrictions, shelter-in-place measures, and travel bans.

In late January, reported COVID-19 cases rose steeply in Hubei Province, and imported cases sparked outbreaks in many other cities throughout China. By February 14, 2020, the government had limited the movement of >500 million persons across 80 cities, many of which rapidly enacted multiple social distancing orders to slow the local spread of the virus, including restricting nonessential services and public transit (*3*–*6*). Given the substantial economic and societal costs of such measures (*7*), estimates of their effectiveness can serve as critical evidence for intervention policy decisions worldwide (*8*).

Using case data from online reports published by the Chinese Center for Disease Control and health commissions (Appendix Table 4), we estimated the time elapsed between the first reported case in a city and successful containment of the outbreak (χ). Technically, we consider an outbreak contained when the 95% CI of the instantaneous reproduction number (R_t_) drops below 1. We analyzed the speed of COVID-19 containment for 58 cities in mainland China outside of Huebei Province that had >20 confirmed cases by February 14, 2020 (Figure; Appendix Tables 2, 3). Collectively, these cities deployed 7 different types of interventions over the course of their epidemics (*9*): bans on entertainment and public gatherings; broad restrictions on public service including healthcare, schooling, shopping, and restaurants; initiation of a level 1 response entailing systematic testing and isolation of confirmed cases; suspension of intracity public transport; suspension of travel between cities; reporting of confirmed cases; recruitment of governmental staff and volunteers to enforce quarantine and social distancing. The mean (+ SD) time between the first confirmed case and the implementation of the first social distancing measure was 13 (+ 4.7) days. By the time these measures were enacted, the median cumulative reported cases in a city was 40, but the range was 9–248 across the 58 cities. The mean time until successful containment was 21 (+ 7) days after the first reported case and 8 (+ 6.8) days following the initiation of interventions. During the period of containment, the reproduction number (R*_t_*) declined by an average of 54.3% (+ 17.6%) (Appendix Figure 2).

**Figure:**
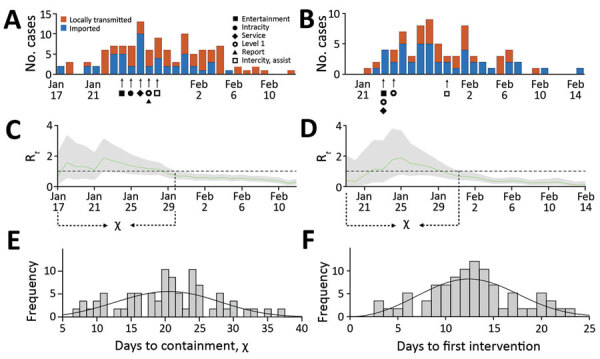
Coronavirus disease (COVID-19) introductions, transmission, and containment for 2 provincial capitals, China, before February 15, 2020. A) Estimated daily incidence of COVID-19 cases and the implementation of local social distancing measures in Xi’an. B) Estimated daily incidence of COVID-19 cases and the implementation of local social distancing measures in Nanjing. C, D) Estimated daily time-varying reproduction numbers (R*_t_*). Green line indicates the median and gray shading 95% CI for R*_t_*. We calculated the number of days from the first reported imported case until the upper 95% CI drops below 1 (χ) for (C) Xi’an and (D) Nanjing. E) The distribution of χ across 58 cities in mainland China. Mean duration of outbreaks is 21 days (SD + 7). Based on an area under the curve comparison between gamma, log-normal, and Weibull distributions fitted via maximum-likelihood to the data, we found that the χ values are roughly Weibull distributed with scale 22.94 (95% CI 21.12–24.91) and shape 3.28 (95% CI 2.68–4.02), indicated by black line. F) The distribution of time between the first locally reported case and the first social distancing measure resembles a Weibull distribution with scale 14.24 (95% CI 13.01–15.60) and shape 2.98 (95% CI 2.44–3.65).

Using a combination of linear regression and best-subsets model selection (*10*), we found that the timing of the first intervention and the initiation of level 1 response significantly predicted the speed of containment across the 36 cities that deployed all 7 interventions (*R*^2^ = 0.27; p<0.001) (Appendix Figure 1). A delay of 1 day in implementing the first intervention is expected to prolong an outbreak by 2.41 (95% CI 0.96–3.86) days. In contrast, the timing of the level 1 response was inversely related to the speed of containment. Level 1 responses were initiated by the central government across mainland China over the course of 1 week, starting with the hardest hit areas in and near Hubei Province on the first day and working outwards toward more distant cities. Thus, the day of level 1 initiation within this 1-week period is a likely indicator for the initial severity of an outbreak and the corresponding difficulty of containment.

We have estimated the value of proactive social distancing interventions in terms of a reduction in days until successful containment. However, because most cities implemented multiple measures quickly and simultaneously, we are unable to disentangle the efficacies of individual modes of social distancing. We note that our estimates of R*_t_* may be biased by the limited case report data available before February 14, 2020; we lack information about testing rates and priorities in China before February 14. As public health agencies around the globe struggle to determine when to implement potentially costly social distancing measures, these estimates highlight the potential long-term benefits of early and decisive action.

Appendix Additional information about effects of proactive social distancing on COVID-19 outbreaks in 58 cities in China.

## References

[1] Chen S, Yang J, Yang W, Wang C, Bärnighausen T. COVID-19 control in China during mass population movements at New Year. Lancet. 2020;395:764–6. 10.1016/S0140-6736(20)30421-9 32105609 PMC7159085

[2] World Health Organization. Coronavirus disease 2019 (‎COVID-19)‎: situation report 80. 2020 [cited 2020 Apr 9]. https://www.who.int/docs/default-source/coronaviruse/situation-reports/20200409-sitrep-80-covid-19.pdf

[3] Chan JF-W, Yuan S, Kok K-H, To KK-W, Chu H, Yang J, et al. A familial cluster of pneumonia associated with the 2019 novel coronavirus indicating person-to-person transmission: a study of a family cluster. Lancet. 2020;395:514–23. 10.1016/S0140-6736(20)30154-9 31986261 PMC7159286

[4] Kraemer MUG, Yang C-H, Gutierrez B, Wu C-H, Klein B, Pigott DM, et al. The effect of human mobility and control measures on the COVID-19 epidemic in China. Science. 2020 Mar 25 [cited 2020 Mar 26]. https://science.sciencemag.org/content/early/2020/03/25/science.abb4218 10.1126/science.abb4218 PMC7146642 32213647

[5] Chinazzi M, Davis JT, Ajelli M, Gioannini C, Litvinova M, Merler S, et al. The effect of travel restrictions on the spread of the 2019 novel coronavirus (COVID-19) outbreak. Science. 2020;368:395–400. 10.1126/science.aba9757 32144116 PMC7164386

[6] Wu Z, McGoogan JM. Characteristics of and important lessons from the coronavirus disease 2019 (COVID-19) outbreak in China: summary of a report of 72,314 cases from the Chinese Center for Disease Control and Prevention. JAMA. 2020;323:1239–42. 10.1001/jama.2020.2648 32091533

[7] Ayittey FK, Ayittey MK, Chiwero NB, Kamasah JS, Dzuvor C. Economic impacts of Wuhan 2019-nCoV on China and the world. J Med Virol. 2020;92:473–5. 10.1002/jmv.25706 32048740 PMC7166799

[8] Leung K, Wu JT, Liu D, Leung GM. First-wave COVID-19 transmissibility and severity in China outside Hubei after control measures, and second-wave scenario planning: a modelling impact assessment. Lancet. 2020;395:1382–93. 10.1016/S0140-6736(20)30746-7 32277878 PMC7195331

[9] Tian H, Liu Y, Li Y, Wu C-H, Chen B, Kraemer MUG, et al. An investigation of transmission control measures during the first 50 days of the COVID-19 epidemic in China. Science. 2020;368:638–42. 10.1126/science.abb6105 32234804 PMC7164389

[10] Yang H. The case for being automatic: introducing the automatic linear modeling (LINEAR) procedure in SPSS statistics. Multiple Linear Regression Viewpoints. 2013;39:27–37.

