# Supplementary material for: Effects of Proactive Social Distancing on COVID-19 Outbreaks in 58 Cities, China
Source: Emerg Infect Dis. 2020 Sep;26(9):2267–9. doi: 10.3201/eid2609.201932 (PMC7454087; doi:10.3201/eid2609.201932)
Supplement: Appendix — Additional information about effects of proactive social distancing on COVID-19 outbreaks in 58 cities in China. [file 20-1932-Techapp-s1.pdf]

Article DOI: https://doi.org/10.3201/eid2609.201932

Effects of Proactive Social Distancing on COVID-19 Outbreaks in 58 Cities, China

Appendix

Supplemental Methods

We collected data from online reports published by China Center for Disease Control and Prevention and health commissions (Appendix Table 4). The data comprised 8,410 confirmed cases before February 15, 2020, across 271 cities in mainland China with known dates of symptom onset and classified as imported or locally infected. In any case, with missing symptom onset timings, we allocate a random value post the official announcement following the gamma distribution of the period from the onset of symptoms (*1*). We also aggregated data on the timing of 7 different classes of social distancing measures in 58 cities outside Hubei province (Appendix Table 1; Appendix 2).

Estimation of R_t

We estimate the time-varying reproduction numbers for the outbreaks in 58 cities using the R package EpiEstim (*2*) based on the method of Ref (*3*). Given time series data for incident imported and locally-infected cases and the distribution of serial intervals, the algorithm produces the time series of R_t with medians and 95% CI. We assume the serial interval follows the gamma distribution (mean 5.11 days, SD 2.68 days (*1*) and the length of sliding time window for an estimated 7 days (*4*).

Regression Analysis

To assess the impact of intervention type and timing on the speed of containment, we applied a simple regression and variable selection method (Appendix Table 2). Specifically, we fit a linear regression model predicting the time until containment (i.e., days between symptom onset for the first reported case and the estimated 95% CI upper bound of R_t dropping below 1) as given by

$$\chi = \sum_{i=1}^{8} \beta_i T_i + \epsilon$$

where β_i is the coefficient of the *i*th variable T_i and ϵ is the intercept.

We analyze data from a subset consisting of 36 of 58 cities in mainland China that implemented all 8 interventions and contained the local outbreak by February 14, 2020. We used the best-subsets regression to identify the best fit model for all models containing *k*, in which *k* range is 1–8, and chose our final model based on the Akaike information criterion corrected for small sample sizes (*5*).

Appendix Table 1. Variable definitions for analysis of the mitigating effect of social distancing on coronavirus spread in cities in China, 2020

Symbol	Definition	Candidate model predictor
t_0	Day of symptom onset for the first reported case	No
t_e	Day on which the earliest social distancing measure(s) was enacted	No
$t_{control\ 95}$	Day that the upper 95% CI bound of R_t drops below one (without rebounding)	No
χ	Containment period: the number of days between symptom onset of the first reported case (t_0) and the upper 95% CI of R_t dropping below 1 ($t_{control\ 95}$)	No
T_{SD}†	Days between first reported case (t_0) and implementation of the first social distancing measure implemented (t_e)	Yes
$T_{entertainment}$	Days between the first reported case (t_0) and ban on entertainment and public gatherings (e.g., bar, café, cinema)	Yes
$T_{service}$	Days between the first reported case (t_0) and restrictions on public services, including hospitals, schools, stores, and restaurants	Yes
$T_{level\text{-}1}$‡	Days between the first reported case (t_0) and the initiation of urban level-1 response for systematic testing and isolation of confirmed cases	Yes
T_{intra_trans}	Days between the first reported case (t_0) and suspension of intracity public transport (bus and subway)	Yes
T_{inter_trans}	Days between the first reported case (t_0) and suspension of inbound and outbound travel (i.e., intercity rail, highway, and air travel)	Yes
T_{report}	Days between the first reported case (t_0) and online posting of confirmed case reports	Yes
T_{assist}	Days between the first reported case (t_0) and the recruitment of governmental staff and volunteers to assist with quarantine and social distancing	Yes

Appendix Table 2. Results of linear regression relating immediacy of interventions to speed of containment, China*

Predictors	Coefficient	p value
Intercept	14.37 (9.02, 19.72)	0.000
T_{SD}†	2.41 (0.97, 3.86)	0.002
$T_{level\text{-}1}$	−1.87 (−3.14, −0.60)	0.005

*Speed of containment is defined as days until upper 95% CI of R_t <1. We included in the regression results for 36 of 58 cities that implemented all 8 intervention measures and had sufficient data for prediction available (χ) by February 14, 2020. We selected the parameters in the table from among all of the candidates in Appendix Table 1 using the best-subsets method (*6*) that identifies the most informative combinations of predictors with respect to the Hurvich and Tsai's Information Criterion (7). The fitted model has R^2 = 0.27, p<0.001.
†Days between first reported case (t_0) and implementation of the first social distancing measure implemented (t_e).
‡Days between the first reported case (t_0) and the initiation of urban level-1 response for systematic testing and isolation of confirmed cases.

Appendix Table 3. Distribution fits for the time between the first reported case and containment (χ) and time between the first reported case and the implementation of the first social distancing measure*

Time	Distribution	Shape (95% CI)	Scale (95% CI)	Akaike's Information Criterion
χ	Gamma	7.63 (5.343,10.895)	2.694 (1.864,3.893)	394.164
	Lognormal	2.956 (2.854,3.058)	0.389 (0.329,0.476)	398.975
	Weibull	3.282 (2.682,4.016)	22.937 (21.119,24.911)	390.329
T_{SD}	Gamma	6.121 (4.294,8.725)	2.079 (1.437,3.008)	349.986
	Lognormal	2.46 (2.342,2.577)	0.448 (0.379,0.549)	357.869
	Weibull	2.981 (2.436,3.649)	14.244 (13.008,15.597)	344.642

*Data were taken from 58 cities in mainland China before February 15, 2020. Timing is calculated in terms of days from symptom onset of the first reported case in the city. Containment is defined by the first day that the estimated upper 95% CI for Rt permanently drops below 1.

Appendix Table 4. Data used in the analysis, which is also available at Github and can be downloaded from https://github.com/MeyersLabUTexas/Proactive-social-distancing-in-Chinese-cities

City name (English)	City name (Chinese)	Chi	Entertainment	Service	Level-1	Intra_ trans	Inter_ trans	Report	Assist	SD	T0 since Jan. 1, 2020
Sanya	三亚	11	17	21	17	18	21	14	25	14	8
Zhongshan	中山	8	13	13	15	16	18	23	28	13	11
Linyi	临沂	20	17	17	16		18	15	24	15	8
Jiujiang	九江	37	23	23	20	23	26	20	29	20	3
Xinyang	信阳	24	21	21	23	23	30	23	31	21	3
North Sea	北海	16	14		16	23	23	12	24	12	9
Nanjing	南京	20	11	11	12		18	10	30	10	12
Nanning	南宁	30			10			11	23	10	15
Nanchang	南昌	25	15	15	11		15	19	29	11	12
Nanyang	南阳	22	20	20	22	22	22	30	30	20	4
Hefei	合肥	20	14	25	13	15	17	18	24	13	10
Harbin	哈尔滨	21	21	14		22		22		14	13
Shangqiu	商丘	35	22	22		26	26			22	2
Tianjin	天津	28		18	15	23	18	13	24	13	9
Weihai	威海	15	17	17	9	15	12	9	30	9	15
Ningbo	宁波	20	6	6	6	10	10	12	27	6	17
Anqing	安庆	18	15	22	13	16	17	16	28	13	10
Yichun	宜春	25	9	9	9	11	15	9	29	9	16
Suzhou	宿州	30	13	19	12	16	15	15	31	12	12
Yueyang	岳阳	32	20	23	18	23	18	24	26	18	6
Pingdingshan	平顶山	27	14	14	15			20		14	11
Kaifeng	开封	10	3	3	9			5	33	3	21
Zhangjiakou	张家口	24	6	6	7	14	9	11	26	6	18
Xuzhou	徐州	28	17	17	17	20	20	19	30	17	7
Huizhou	惠州	28	16	16	18		20	14	28	14	9
Chengdu	成都	25	20	20	20		20	11	39	11	11
Fuzhou	抚州	24	15	15	15	17	17	15	29	15	10
Xinyu	新余	33	27	27	20		28	29	29	20	3
Wuxi	无锡	22	11	9	9	13	11	8	30	8	15
Hangzhou	杭州	19	16	16	12	21	16	15	27	12	11
Taian	泰安	14	3	3	3	6	6	7	25	3	21
Taizhou	泰州	24	13	13	12	17	17	14	30	12	12
Jinan	济南	23	15	15	15		17	15	23	15	9
Jining	济宁	24	10	10	8	14	10	8	25	8	16
Haikou	海口	24	20	20	16		20	13	25	13	9
Huaian	淮安	21	12	9	5	7	8	7	30	5	19
Shenzhen	深圳	15			23	28	28	28	28	23	1
Wenzhou	温州	22	20	20	19	26	23	17	27	17	4
Weifang	潍坊	20	6	6	6	7	10	9	30	6	18
Zhuhai	珠海	16	13	14	19	25	19	13	28	13	10
Yiyang	益阳	21	21	23	21	25	23	22	29	21	3
Yancheng	盐城	20	18	19	15		20	17	30	15	9
Shijiazhuang	石家庄	18	17	17	14	17	17	12	26	12	10
Shaoxing	绍兴	7	10	10	9	9	13	11	26	9	14
Wuhu	芜湖	8	11		10	17	13	12	26	10	14
Suzhou	苏州	16	12	12	11	15	16	10	30	10	13
Pingxiang	萍乡	25	16	16	16	17	18	16	29	16	10
Quzhou	衢州	15	15	15	14	21	18	14	23	14	9
Xi'an	西安	19	14	17	15	18	16	13	28	13	10
Xuchang	许昌	21	4	6	7	7	7	7	27	4	20
Ganzhou	赣州	29	20	20	19	35	24	26	29	19	6
Zhengzhou	郑州	11	22	22	23	23	23	17	31	17	4
Chenzhou	郴州	11	13	13	12	16	16	11	30	11	12
Jinhua	金华	21	12	12	11	15	15	11	27	11	12
Changchun	长春	13	13	13	13	16	14	11	26	11	12
Fuyang	阜阳	18	15	15	15	23	17	16	29	14	10
Ma'anshan	马鞍山	10	10	20	9	13	12	11	42	9	15
Zhumadian	驻马店	9	12	12	14		21	14	31	12	12

References

1. Zhang J, Litvinova M, Wang W, Wang Y, Deng X, Chen X, et al. Evolving epidemiology and transmission dynamics of coronavirus disease 2019 outside Hubei province, China: a descriptive and modelling study. Lancet Infect Dis. 2020 Apr 2 [Epub ahead of print]. PubMed https://doi.org/10.1016/S1473-3099(20)30230-9

2. Cori A. EpiEstim: Estimate time varying reproduction numbers from epidemic curves. 2019 [cited 2020 Jun 1]. https://cran.r-project.org/web/packages/EpiEstim/index.html

3. Thompson RN, Stockwin JE, van Gaalen RD, Polonsky JA, Kamvar ZN, Demarsh PA, et al. Improved inference of time-varying reproduction numbers during infectious disease outbreaks. Epidemics. 2019;29:100356. PubMed https://doi.org/10.1016/j.epidem.2019.100356

4. Cowling BJ, Ali ST, Ng TWY, Tsang TK, Li JCM, Fong MW, et al. Impact assessment of non-pharmaceutical interventions against coronavirus disease 2019 and influenza in Hong Kong: an observational study. Lancet Public Health. 2020;5:e279–88. PubMed https://doi.org/10.1016/S2468-2667(20)30090-6

5. Hurvich CM, Tsai C-L. Regression and time series model selection in small samples. Biometrika. 1989;76:297–307.

6. Yang H. The case for being automatic: introducing the automatic linear modeling (LINEAR) procedure in SPSS statistics. Multiple Linear Regression Viewpoints. 2013;39:27–37.

7. IBM Knowledge Center. Information Criteria [cited 2020 Mar 30]. https://www.ibm.com/support/knowledgecenter/SSLVMB_23.0.0/spss/tutorials/mixed_diet_info_02.html

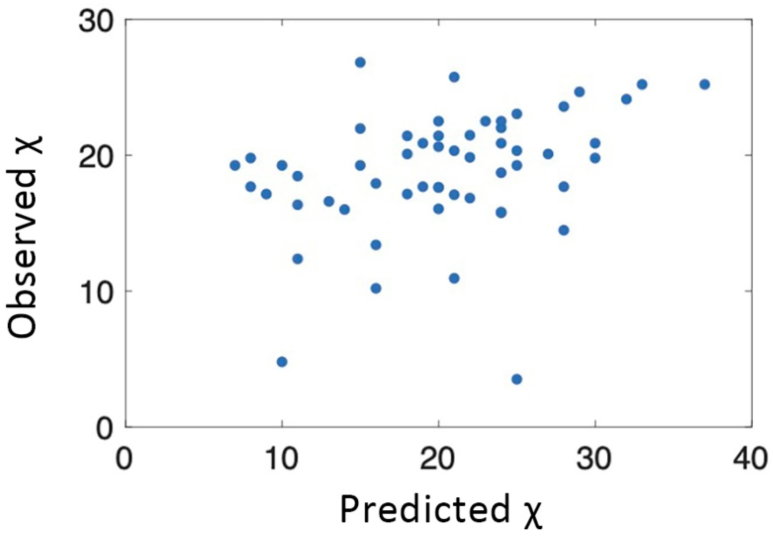

Appendix Figure 1. Predicted versus observed speed of containment (χ) for 36 cities in mainland China based on the fitted regression model (Appendix Table 1). The data have a Pearson correlation coefficient of 0.52 (p<0.001).

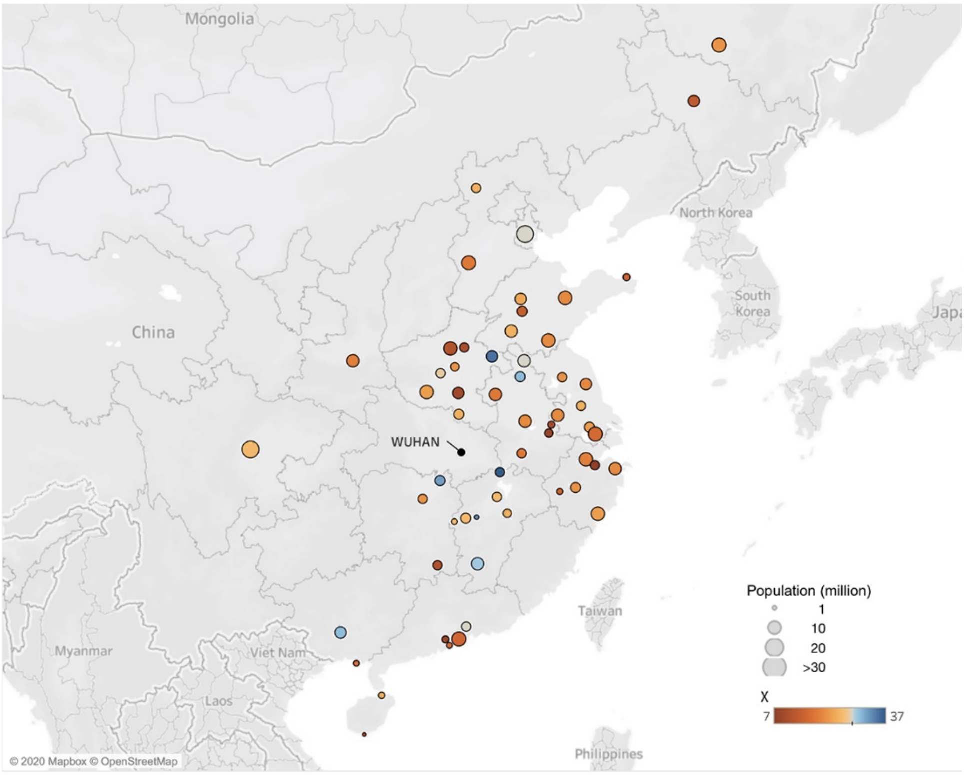

Appendix Figure 2. The number of days between the first reported case and containment (χ) for 58 cities in mainland China that achieved containment before February 15, 2020. Outbreaks are considered contained when the estimated upper 95% CI bound of R_t drops below 1 without rebounding. Orange indicates the 48 cities that achieved containment within 4 weeks; blue indicates the 10 cities that did not.
